# Snowstorm Enhanced the Deterministic Processes of the Microbial Community in Cryoconite at Laohugou Glacier, Tibetan Plateau

**DOI:** 10.3389/fmicb.2021.784273

**Published:** 2022-01-27

**Authors:** Yuying Chen, Yongqin Liu, Keshao Liu, Mukan Ji, Yang Li

**Affiliations:** ^1^State Key Laboratory of Tibetan Plateau Earth System, Resources and Environment (TPESRE), Institute of Tibetan Plateau Research, Chinese Academy of Sciences, Beijing, China; ^2^College of Resources and Environment, University of Chinese Academy of Sciences, Beijing, China; ^3^Center for the Pan-Third Pole Environment, Lanzhou University, Lanzhou, China; ^4^Institute of International Rivers and Eco-Security, Yunnan University, Kunming, China

**Keywords:** cryoconite, snowfall, rare bacteria, deterministic processes, stochastic processes

## Abstract

Cryoconites harbor diverse microbial communities and are the metabolic hotspot in the glacial ecosystem. Glacial ecosystems are subjected to frequent climate disturbances such as precipitation (snowing), but little is known about whether microbial communities in cryoconite can maintain stability under such disturbance. Here, we investigated the bacterial community in supraglacial cryoconite before and after a snowfall event on the Laohugou Glacier (Tibetan Plateau), based on Illumina MiSeq sequencing of the 16S rRNA gene. Our results showed that the diversity of the microbial community significantly decreased, and the structure of the microbial community changed significantly after the disturbance of snowfall. This was partly due to the relative abundance increased of cold-tolerant bacterial taxa, which turned from rare into abundant sub-communities. After snowfall disturbance, the contribution of the deterministic process increased from 38 to 67%, which is likely due to the enhancement of environmental filtering caused by nitrogen limitation. These findings enhanced our understanding of the distribution patterns and assembly mechanisms of cryoconite bacterial communities on mountain glaciers.

## Introduction

Cryoconite is dark sediment deposited on the glacial surfaces, comprising both mineral and biological materials. Supraglacial cryoconite serves as a habitat for diverse microbial communities, including viruses, bacteria, fungi, archaea, algae, and invertebrates, which are responsible for the glacier biogeochemical cycling (Anesio et al., 2007, 2010; Edwards et al., 2011, 2013; Zarsky et al., 2013). Supraglacial cryoconite microbes are directly exposed to environmental pressures or perturbations, such as frequent flushing by supraglacial meltwater and precipitation (Zawierucha et al., 2019b). Thus, understanding the response of microbial communities to climate disturbance is essential for the study of ecosystem functions (Steudel et al., 2012).

Microbial communities are affected by various physicochemical factors (Stibal et al., 2012; Cook et al., 2016; Anesio et al., 2017). Temperature and nutrient availability have been proposed to be the main factors controlling the microbial communities in polar and mountain glacier cryoconite (Zarsky et al., 2013; Ambrosini et al., 2016; Liu et al., 2017; Sommers et al., 2017; Lutz et al., 2018). Differences in physicochemical conditions can also indirectly influence microbial community structure in cryoconite by altering biotic interactions (Friedman and Gore, 2017; Khan et al., 2018; Bergk Pinto et al., 2019). For example, on Spitsbergen Island in Svalbard, the addition of organic carbon shifted microbial interactions from collaboration to competition (Bergk Pinto et al., 2019). Intensive collaboration can enhance complex organic carbon degradation and mineralization, which are particularly important for oligotrophic environments such as glaciers (Bergk Pinto et al., 2019; Krug et al., 2020). These interactions change can favor or discriminate certain microbial groups, thereby alter the microbial community structure (i.e., biofiltering).

Recently, a growing body of research has emphasized the ecological importance of rare taxa (Lynch and Neufeld, 2015; Jousset et al., 2017), and distinct succession patterns and functional characteristics have been found between the abundant and rare taxa (Jia et al., 2018; Jiao and Lu, 2020; Rocca et al., 2020). The abundant taxa are usually perceived to be involved in the active category in biogeochemical cycles, especially carbohydrate metabolism, and take up the core ecological niches (Jiao et al., 2017; Kurm et al., 2019; Liang et al., 2020). In addition, due to their high abundance, the abundant taxa might present greater tolerance to environmental stresses (Jiang et al., 2019; Jiao and Lu, 2020). The rare taxa serve as a reservoir of genetic and functional diversity for the entire community and contribute to the maintenance of microbial diversity (Jousset et al., 2017; Rocca et al., 2020). That is, when facing environmental disturbance, rare taxa could respond promptly to maintain community stability (Zhang et al., 2020). For example, some rare taxa may turn into dominant taxa in the community to compensate for the ecosystem function loss due to the disturbance (Jiang et al., 2019; Du et al., 2020). The interaction and transformation of abundant and rare bacterial are critical for maintaining functional redundancy and community stability (Liang et al., 2020). Therefore, additional studies are needed to investigate the relationship between abundant and rare bacterial groups in cryoconite communities after disturbance.

Previous studies have demonstrated that two different assembly mechanisms, stochastic and deterministic processes, affect abundant and rare bacterial sub-communities in different patterns. Deterministic processes include niche-based factors such as abiotic and biotic factors (Elshahed et al., 2008; Pedros-Alio, 2012; Sauret et al., 2014). Stochastic processes include probabilistic dispersal and random changes in the relative abundance of species (ecological drift) (Liu et al., 2015; Zawierucha et al., 2019a,b). Little is known about stochastic or deterministic processes structuring the rare and abundant microbial communities in the cryoconite ecosystem. A recent study found that stochastic weather events (rainstorms) changed the tardigrades community structure on an Arctic glacier (Zawierucha et al., 2019b). The authors also reported that the tardigrades in the Arctic cryoconite granules were passively dispersed by streams and melting water, while pH and electrical did not shape invertebrate communities (Zawierucha et al., 2019b). However, whether the community assembly of cryoconite microbial communities is affected by climate disturbance remains largely unknown.

Here we studied the changes and assembly mechanisms of microbial communities in supraglacial cryoconite before and after a snowfall event in the Laohugou Glacier (LHG), Tibetan Plateau. The Tibetan Plateau contains the largest area of glacial ice outside the polar regions and has an average altitude > 4,000 m above sea level (a.s.l) (Yao et al., 2012). Compared to other areas with alpine glaciers, the glaciers in the Tibetan Plateau are exposed to lower temperatures and lower allochthonous inputs of organic matter, due to the higher altitude (Liu et al., 2017). The aims of the present study are: (i) to explore the cryoconite microbial community structure and assembly process following snowfall disturbance; (ii) to investigate the response of the abundant and rare bacterial sub-communities after disturbance.

## Materials and Methods

### Sites and Sampling

The LHG (39°26.4′N, 96°32.5′E), with a length of 10.1 km and an area of 21.9 km^2^, is the largest glacier in the Qilian Mountains, in the northeastern Tibetan Plateau (Sun et al., 2012). It is a typical valley glacier, with elevations ranging from 4,200 m a.s.l at its terminus to 5,481 m a.s.l at its top. The region has a typical continental climate affected by desertification in central Asia, with an average temperature above 0°C in summer and long periods below freezing in winter. Precipitation is mainly concentrated in May-September, primarily affected by westerly winds (Wei et al., 2017).

In August 2016, we randomly selected four superficially deposited cryoconite mounds, which piled up due to glacial melting within the ablation zone for collecting samples (Supplementary Figure 1). Four sampling sites were 100–400 m apart, covering a large area in order to represent the glacier’s features. To ensure that collecting was made at the same locations, we determined the coordinates of each site using GPS and mounted bamboo sticks in the ice. Samples were collected from each of the observation sites on the 1st, 4th, 8th, 26th, and 31st of August, which are referred as day 1, 4, 8, 26, and 31 hereafter). The large gap between the third and fourth sampling days was due to heavy snowfall and flooding that occurred after 10th August. Before sampling, we scraped off the surface layer (top 1 cm) of cryoconite. Approximately 200 g of cryoconite debris were collected using a pre-cleaned stainless-steel spoon with polyethylene gloves worn all the time. The sample was placed into sterile 250 ml Nalgene ^®^ plastic bottles that were first cleaned with tap water and then rinsed three times with Milli-Q water, followed by sterilization for 15 min at 120°C in steam autoclaving and drying at 55°C. Samples were transported in a dark container within 4 h to the Qilian Shan Station of Glaciology and Ecologic Environment, Chinese Academy of Sciences. All samples were kept frozen at −20°C until laboratory analysis.

### Geochemical Measurements

The total organic carbon (TOC) contents of the samples were determined using a TOC-L (Shimadzu Corp., Kyoto, Japan) on lyophilized samples. Total nitrogen (TN) and total carbon (TC) were measured using an elemental analyzer (Vario MAX, Elementar, Germany). The elemental concentrations are reported as the percentage of dry weight (100°C for 24 h) of cryoconite material.

### DNA Extraction and Illumina Sequencing

Cryoconite debris (0.5 g) was soaked in 1.5 mL of lysis buffer (20 mg mL^–1^ Proteinase K, 0.1 M EDTA, and 10% SDS) and incubated at 55°C for 2 h before DNA extraction. DNA extraction was performed using a Fast DNA ^®^SPIN Kit for Soil (MP Biomedicals, Santa Ana, CA, United States), according to the manufacturer’s instructions. The extracted DNA was eluted to 100 μL TE buffer. The raw DNA was purified using a 1% (w/v) agarose gel. DNA bands were removed from the gel and extracted using an Agarose Gel DNA purification kit (TaKaRa, Japan). The concentration of DNA extracts was measured by the NanoDrop 1000 spectrophotometer (Thermo-Scientific, Wilmington, DE, United States). The extracted DNA was stored at -80°C until amplification.

The V4 region of the bacterial 16S rRNA genes was amplified in triplicate by primer pair 515F (5′-GTGCCAGC MGCCGCGGTAA-3′) and 806R (5′- GGACTACHVGGGTW TCTAAT-3′) and sequenced on an Illumina MiSeq system (Illumina, Inc., San Diego, CA, United States) following the procedure by Caporaso et al. (2012). Briefly, PCR was performed under the condition of 94°C for 5 min, 30 cycles of 94°C for 30 s, 52°C for 30 s, 72°C for 30 s; followed by a final cycle of 10 min at 72°C. Each PCR reaction contained 25 μL 2x Premix Taq DNA polymerase (Takara Biotechnology, Dalian Co., Ltd., China), 3 μL DNA template (20 ng μL^–1^), 1 μL each primer (10 μM), and 20 μL nuclease-free water. PCR products of the triplicate reaction samples were pooled and quantified by PicoGreen staining. The sample libraries were generated with purified PCR products. The MiSeq 500 cycles kit was used for 2 × 250 bp paired-end sequencing.

### Processing of Illumina Sequencing Data

The “fastq_filter” command in USEARCH was used to trim and filter the raw sequence reads (Edgar, 2016). The trimmed and filtered reads were used to call amplicon sequence variants (ASVs) using the “-cluster_ASVs” and “-unoise3” commands in USEARCH, respectively (Edgar, 2016). Singleton ASVs with fewer than nine reads were removed using the default “-minsize” values. The “-usearch_global” command with a minimum sequence identity of 99% was used to map ASVs sequences. Chimeric ASVs were removed using the “uchime_ref” command in USEARCH. Representative sequences were taxonomically classified using the “sintax” command along with the RDP training set (version 16) with confidence scores above the 0.5 cutoff (Chiellini et al., 2019). Normalization across samples was performed using a variance stabilizing transformation with the DESeq2 package in R (McMurdie and Holmes, 2014). After the taxonomy assignment, ASVs associated with chloroplasts, archaea, and unclassified sequences were excluded from subsequent analyses.

### Definition of Abundant and Rare Taxa

Rare taxa were defined as the ASVs with mean relative abundance < 0.1% in all samples in one group, whereas abundant ASVs were defined as the ASVs with a relative abundance > 1% in one or more samples in one group (Ji et al., 2020; Zhang et al., 2020; Dong et al., 2021). Taxa that did not fall into either the abundant or rare categories were defined as the oscillating taxa.

### Statistical Analysis

Wilcoxon rank-sum test and Kruskal-Wallis test were used to determine whether there were any statistically significant differences among groups of environmental variables. The alpha-diversity indices (Shannon-Wiener and Pielou’s Evenness) were calculated using the “vegan” R package (Oksanen et al., 2010). Wilcoxon rank-sum test and Kruskal-Wallis test were used to determine whether there were any statistically significant differences among groups of alpha-diversity. The significance of dissimilarity of community composition among groups was tested using permutational multivariate analysis of variance (PERMANOVA) based on Bray-Curtis distance metrics with the “adonis” function in the R package “vegan” (Oksanen et al., 2010). Test results with *P* < 0.05 were considered as statistically significant. The correlations between bacterial diversity and environmental variables were calculated using Spearman’s rank correlation, as implemented in the “ppcor” R package. Mantel test based on Spearman’s rank correlations was performed to show the correlation between variations of microbial communities and environmental variables using the “vegan” package of R (Oksanen et al., 2010).

Co-occurrence networks were explored using Molecular Ecological Network Analysis Pipeline (MENA2)^1^ based on the Random Matrix Theory (RMT) and Spearman correlation (Deng et al., 2012). The resulting correlation matrix was analyzed with the RMT-based network approach to determine the correlation threshold for network construction, and the same threshold was used for all networks, so the topological properties of all networks are comparable. Then global network properties were calculated including individual nodes’ centrality, degree, betweenness, and clustering coefficient. Integrating all the results obtained from MENA, co-occurrence network was visualized by Gephi.

The normalized stochasticity ratio (NST) based on the Bray–Curtis dissimilarity was calculated using the “NST” package in R to estimate the determinacy and stochasticity of the bacterial assembly processes with high accuracy and precision (Ning et al., 2019). The NST index used 50% as the boundary point between more deterministic (< 50%) and more stochastic (> 50%) assembly processes.

## Results

### Environmental Characteristics of the Cryoconite Samples

**FIGURE 1:**
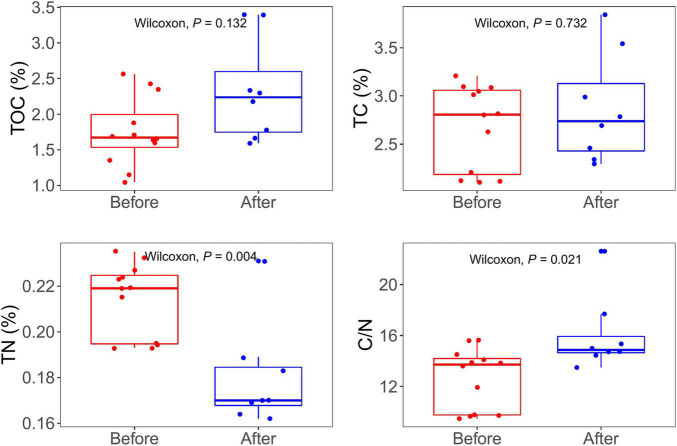
Total dissolve carbon (TOC), total carbon (TC), total nitrogen (TN), and rate of carbon nitrogen (C/N) concentrations in samples before and after snowfall disturb. Each dot represents an individual sample. Significantly higher concentrations of TN were observed in the samples before snowfall, while a significantly higher ratio of C/N was in the samples after snowfall (Wilcoxon rank-sum test, *P* < 0.05).

The TOC concentration ranged from 1.04 to 3.39%. The TC concentration ranged from 2.10 to 3.85%. The concentration of TOC and TC of samples after snowfalls were similar between the samples before and after snowfalls (Wilcoxon rank-sum test, *P* = 0.13, and *P* = 0.73, respectively). The TN concentrations ranged from 0.16 to 0.24%, and that before the snowfall was significantly higher than that after snowfall (Wilcoxon rank-sum test, *P* = 0.003). The C/N ratio ranged from 9.47 to 22.62, and the C/N ratio of samples after the snowfall was significantly higher than those before the snowfall (Wilcoxon rank-sum test, *P* = 0.02; Figure 1). All environmental characteristics showed no significant difference between sampling sites (Kruskal-Wallis test, all *P* > 0.05; Supplementary Figure 2).

### Taxonomic Compositions of Bacterial Community

**FIGURE 2:**
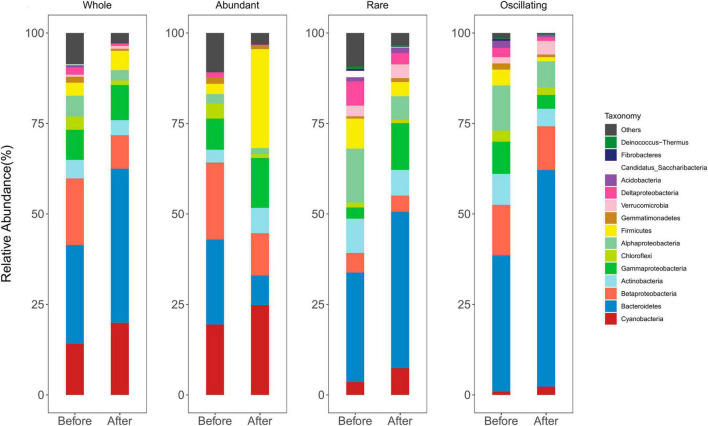
Taxonomic composition of bacteria before and after snowfalls in whole, abundant, and rare communities. The communities were dominated by Cyanobacteria, Bacteroidetes, Betaproteobacteria, Actinobacteria, Gammaproteobacteria, Chloroflexi, Alphaproteobacteria, and Firmicutes.

After quality filtering and the removal of low-quality sequences, a total of 867,658 reads were obtained, and were clustered into 872 ASVs (99% sequence similarity). The most abundant bacterial phyla were Bacteroidetes, Cyanobacteria, Betaproteobacteria, Gammaproteobacteria, Actinobacteria, Alphaproteobacteria, Chloroflexi, and Firmicutes, which account for 86.2 and 95.0% of sequence abundance before and after snowfalls, respectively (Figure 2). The composition of the abundant sub-community was similar to the whole community. However, the rare and oscillating sub-communities differed from the abundant sub-communities. The rare and oscillating taxa contained more taxonomic groups than the abundant taxa. Bacteroidetes, Alphaproteobacteria, Verrucomicobia, Deltaproteobacteria, and Acidobacteria were more prevalent in rare and oscillating sub-communities, while Cyanobacteria and Betaproteobacteria were more prevalent in abundant sub-community (Figure 2).

In the whole community, the relative abundance of Betaproteobacteria, Chloroflexi, Alphaproteobacteria, Gemmatimonadetes, and Deltaproteobacteria were significantly higher before snowfall than after snowfalls (Wilcoxon rank-sum test, all *P* < 0.05; Supplementary Figure 3). In the abundant sub-community, the relative abundance of Bacteroidetes, Chloroflexi, Alphaproteobacteria, and Deltaproteobacteria were significantly higher before snowfall than after snowfalls (Wilcoxon rank-sum test, all *P* < 0.05), while Actinobacteria were significantly higher after snowfall than before snowfalls (Wilcoxon rank-sum test, *P* < 0.01). In the rare sub-community, the relative abundance of Alphaproteobacteria and Deltaproteobacteria were significantly higher before snowfall than after snowfalls (Wilcoxon rank-sum test, all *P* < 0.05). In the oscillating sub-community, the relative abundance of Gammaproteobacteria, Firmicutes, Gemmatimonadetes, and Deltaproteobacteria were significantly higher before snowfall than after snowfalls (Wilcoxon rank-sum test, all *P* < 0.05; Supplementary Figure 3).

The abundant taxa (34-48 ASVs) accounted for 62.1–94.5% of all sequences. The rare taxa (167-495 ASVs) only accounted for 2.6–13.6% of all sequences. The oscillating taxa (40-128 ASVs) accounted for 2.2–29.4% of all sequences (Supplementary Table 1). After snowfall disturbance, the proportion of abundant bacterial taxa did not change significantly, while the percentage of rare taxa significantly increased, and the percentage of oscillating taxa significantly decreased (Supplementary Figure 4). Approximately 59% of abundant bacterial taxa before snowfall were still abundant after the snowfall, and 8% became rare bacterial taxa, and 35% became oscillating bacterial taxa. After the snowfall disturbance, a large proportion (93%) of rare bacterial taxa still being the rare bacterial taxa, while 1% became abundant bacterial taxa, and 1% became oscillating bacterial taxa, and an additional 5% disappeared. Only 29% of oscillating bacterial taxa in samples before snowfall were still oscillating in bacterial taxa after the snowfall, 2% became abundant taxa, and 69% became rare bacterial taxa (Supplementary Table 2). The abundant bacterial taxa that came from the rare and oscillating bacterial taxa after snowfall accounted for 23 and 19% of the community. They are primarily taxonomically classified as *Flavobacterium* (Bacteroidetes), *Massilia* (Betaproteobacteria), *Polaromonas* (Betaproteobacteria), *Psychrobacter* (Gammaproteobacteria), *Cellulomonas* (Actinobacteria), and *Carnobacterium* (Firmicutes) (Supplementary Table 3).

### Diversity of Bacterial Community

**FIGURE 3:**
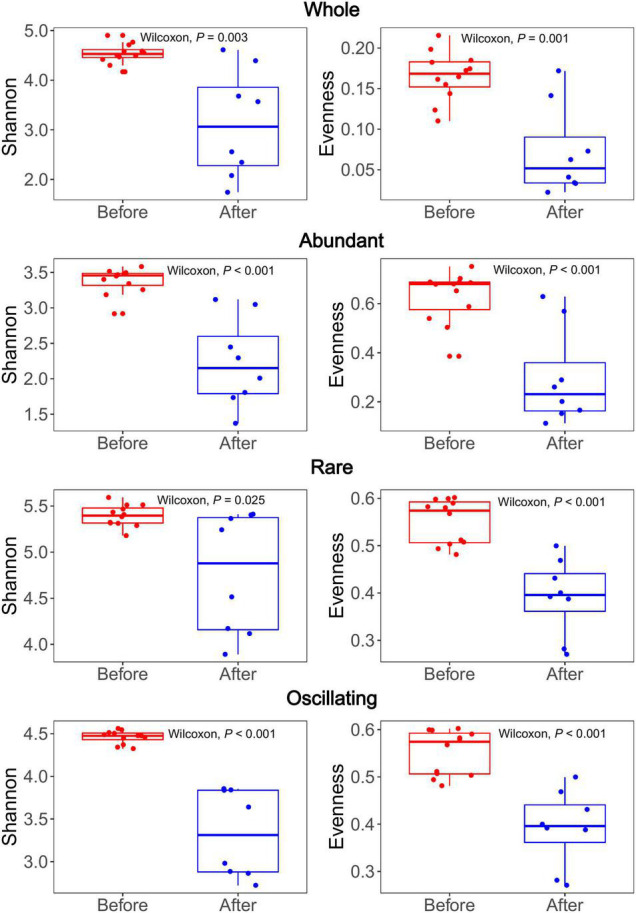
Bacterial alpha diversity comparison between the communities before and after snowfall. Each dot represents an individual sample. For both Shannon and Evenness indices, significant higher diversity was observed in communities before snowfall in the whole, abundant, rare and oscillating communities (Wilcoxon rank-sum test, *P* < 0.05).

The Shannon-Wiener diversity of the whole, abundant, rare, and oscillating community were 1.74–4.90, 1.47–3.67, 3.79–5.38, and 2.72–4.56, respectively. The Pielou’s Evenness of the whole, abundant, rare, and oscillating community were 0.02–0.22, 0.07–0.60, 0.46–0.68, and 0.33–0.85, respectively. The Shannon diversity indices of the rare sub-community were significantly higher than those of the abundant and oscillating sub-communities (Wilcoxon rank-sum test, all *P* < 0.001; Supplementary Figure 5). After the snowfall, the Shannon-Wiener and Pielou’s Evenness indices were significantly reduced in all subcommunities (Wilcoxon rank-sum test, all *P* < 0.05; Figure 3). The Shannon and Evenness indices positively correlated with the TN concentration (Spearman’s correlation: *r* = 0.45, *P* < 0.05; *r* = 0.45, *P* < 0.05; Table 1).

**TABLE 1:** Correlation between cryoconite community Shannon and Evenness indices and environmental factors.

Environmental factors	Shannon	Evenness
TOC	−0.30	−0.27
TC	−0.11	0.00
TN	**0.45**	**0.45**
C/N ratio	−0.33	−0.25

*Significant correlations (at P < 0.05) are in bold.*

**FIGURE 4:**
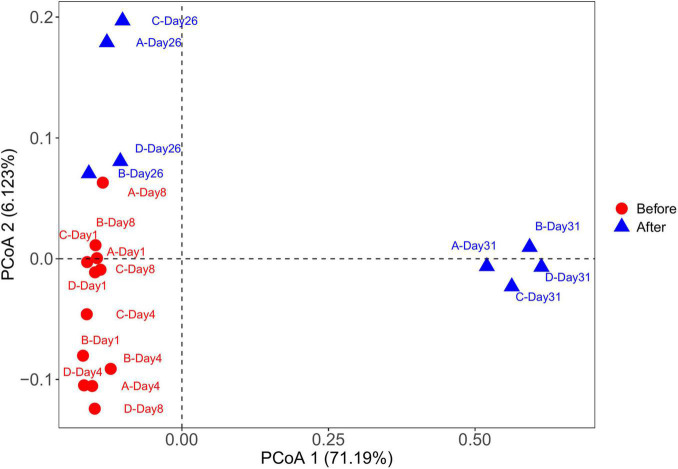
Principal coordinate analysis (PCoA) of microbial communities from cryoconite samples based on Bray-Curtis distance. The PCoA plot showed clear segregation between the before and after snowfall samples.

The PCoA plot showed clear segregation between the samples before and after snowfall (Figure 4). The result was also confirmed by the PERMANOVA analysis (PERMANOVA: *F* = 7.57, *P* < 0.001). Mantel tests revealed significant correlations of the community composition with the TN concentration (Mantel test: *r* = 0.45, *P* = 0.002; Table 2).

**TABLE 2:** Correlation relationships between the community compositional dissimilarity and the measured environmental factors by Mantel test.

Environmental factors	Whole community	
	R	*P*
TOC	0.04	0.369
TC	0.19	0.071
TN	**0.45**	0.002
C/N ratio	0.12	0.241

*Significant correlations (at P < 0.05) are in bold.*

### Co-occurrence Patterns of Bacterial Community

**FIGURE 5:**
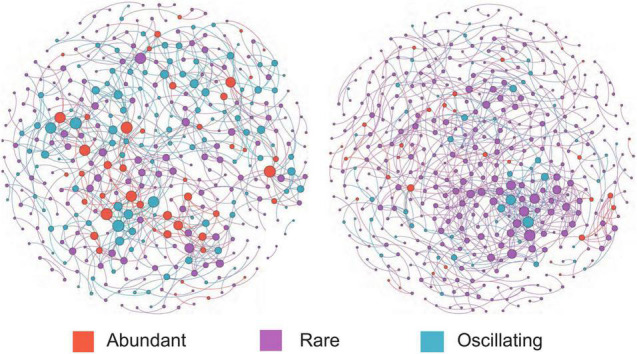
Bacterial Co-occurrence networks for the communities before and after snowfall. Each node represents an amplicon sequence variant (ASV). Nodes are colored by abundant, rare, and oscillating taxa, and the node sizes were proportional to their node degrees. The community network after snowfall is more complex with a higher connectedness than before snowfall.

The co-occurrence network of bacterial communities was performed at the ASV level based on Spearman’s correlation relationships to examine the interactions between microorganisms. The co-occurrence network of bacterial communities was more complex after snowfall (Figure 5). Before the snowfall, the network contained 379 nodes which were connected by 690 edges, while after the snowfall the network contained 454 nodes and 726 edges. In addition, the networks before and after snowfall had 675 and 692 positive edges, respectively, but only 15 and 34 negative edges, respectively. The network after snowfall showed higher values of module number (68) and modularity (0.84) than the network before snowfall (31 and 0.82, respectively), indicating increased degree of modularity (Table 3). The nodes in the network before snowfall were composed of abundant ASVs (12.0%), rare ASVs (57.6%), and oscillating ASVs (30.4%). The nodes in the post snowfall network were composed of a lower proportion of abundant and oscillating ASVs (7.0 and 11.7%), but a higher proportion of rare ASVs (81.3%).

**TABLE 3:** Topological properties of the before and after snowfall microbial networks.

	Before	After
Nodes	379	454
Edges	690	726
Positive edges	675 (98%)	692 (95%)
Negative edges	15 (2%)	34 (5%)
Module number	31	68
Modularity	0.81	0.84

**FIGURE 6:**
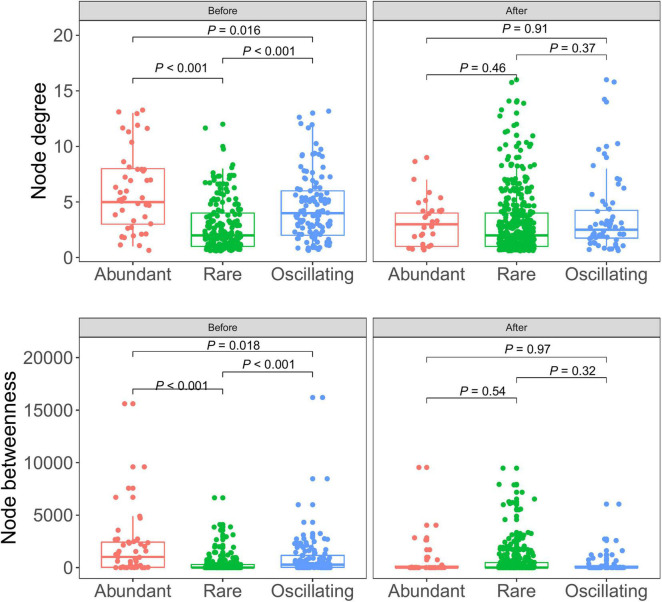
Node-level topological features between abundant, rare, and oscillating taxa in cryoconite communities before and after snowfall disturb. Each dot represents an individual sample. In the community before snowfall, the node degree and betweenness of the abundant bacterial were significantly higher than that of rare bacterial and oscillating taxa (Wilcoxon rank-sum test, *P* < 0.05).

In the community before snowfall, the node degree and betweenness of the abundant bacterial were significantly higher than that of rare bacterial and oscillating taxa (Figure 6), indicating that more information may be passed through abundant bacterial taxa. While in the community post snowfall, no significant differences were detected between abundant, rare and, oscillating taxa.

### Community Assembly Processes

**FIGURE 7:**
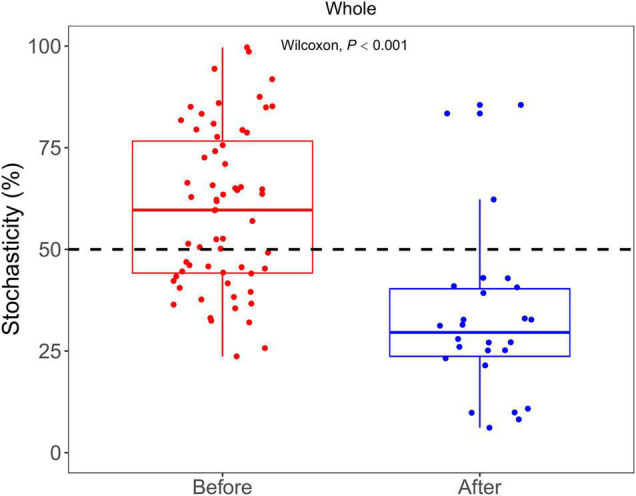
Bar plots show the comparison of NST between the bacterial communities before and after snowfall. The contribution of stochasticity was higher than 50% in the community before snowfall, indicating that the stochasticity process dominates the community assembly processes. The contribution of stochasticity was lower than 50% in the community after snowfall, indicating that the determinism process dominates the community assembly processes. The contribution of stochasticity in cryoconite communities was significantly decreased after snowfall disturbance.

NST was used to examine the relative contributions of stochasticity and determinism in shaping bacterial communities. The average NST values were 62 and 33% in the communities before and after snowfall (Figure 7). After the snowfall, the stochasticity processes significantly decreased for the whole community (Wilcoxon test, *P* < 0.001), suggesting the deterministic processes increased after snowfall disturbance.

## Discussion

### Nitrogen Drives the Bacterial Community Diversity and Community Structure

Bacterial diversity reduced significantly after snowfall disturbance, indicating that the cryoconite microbial community may be subjected to greater environmental filtering after snowfall. This is mostly likely due to nitrogen limitation, as nitrogen was the only measured environmental factors whose concentration significantly reduced post snowfall. This was consistent with the positive correlative relationships between nitrogen and bacterial diversity (Table 1). Nitrogen is an essential nutrient for microbial growth and plays important roles in controlling microbial diversity and ecosystem productivity (Vitousek et al., 2002; Xia et al., 2008; Sun et al., 2014). In the present study, the nitrogen concentration significantly reduced after the snowfall (Figure 1), which could be due to the snowmelt, which lead nitrogen to be flushed out. Generally, the rate at which chemical compounds are released at snowmelt depends on the way a chemical is incorporated in the snow grain (Pomeroy et al., 2005). Important microbial nutrients, such as NO_3_^–^, are not incorporated into the snow grain structure and are released easily (Tranter et al., 1986). The positive correlations between nitrogen availability and alpha diversity indices suggest that nitrogen limitation is an important determinant of bacterial diversity. Similar conclusions have been reported in three glaciers on Tibetan Plateau that TN concentrations were significantly correlated with the alpha diversity (Liu et al., 2017).

Consistent with the alpha-diversity indices, the bacterial community changed after snowfall disturbance (Figure 4), again indicating greater environmental filtering effect. The PCoA results showed that the samples on the 26th day were more similar to those before snowfall, while the samples on the 31st day were significantly different. This may be due to a delayed response to disturbance of the microbial community in cryoconite samples. This was also consistent with the increased deterministic processes in bacterial community structure after snowfall (Figure 7). Increased determinism is frequently attributed to the enhanced environmental filtering (Stegen et al., 2012). Our results demonstrated that TN significantly affected the bacterial community (Table 2). This is consistent with the previous findings that nitrogen availability strongly regulates microbial community structure in cryoconite and ice samples from Arctic and Tibetan Plateau glaciers (Christner et al., 2003; Mueller and Pollard, 2004; Edwards et al., 2011; Cameron et al., 2012; Liu et al., 2017). For example, a prior study indicated that nitrite and nitrate concentrations had important effects on the microbial community local scale variations in cryoconite on the Canada Glacier (Antarctic) (Mueller and Pollard, 2004).

### Nitrogen Limitation Increases Network Complexity

Biotic interactions can explain a substantial proportion of the community structure variations (Hacquard et al., 2015; Dang and Lovell, 2016). A previous study of cryoconite from the High Arctic Foxfonna ice cap found that the assembly of the bacterial community of cryoconite was driven principally by biotic filtering (Gokul et al., 2016). Our results indicated that the community network was more complex after the snowfall disturbance, as evidenced by the higher nodes and edge numbers (Table 3). This is likely due to the enhanced environmental filtering, which has been observed in other systems subjected to nitrogen limitation (Wang et al., 2018). Positive correlations were substantial components in the networks before and after the snowfall (Table 3). Positive relationships could be used to infer commensal, mutualistic, obligate syntrophic, and parasitic relationships between bacterial species (Ji et al., 2019). The increased negative correlations after snowfall disturbance may be due to nutrient competition caused by nitrogen limitation (Zhao et al., 2016). It was proposed that the relationship between microorganisms occupying a similar ecological niche could change from commensalism (positive correlations) to competition (negative correlations) when nutrients become limited (Ji et al., 2019). Thus, the nitrogen limitation may influence bacterial diversity and community structure directly and indirectly.

### Rare Bacteria Play an Important Role in the Microbial Community

The rare sub-community possessed a higher diversity than the abundant sub-community (Supplementary Figure 5). Thus, the rare taxa are the reservoir of genetic seeds to maintain community diversity and stability (Lennon and Jones, 2011; Rocca et al., 2020), providing biological buffering capacity to withstand environmental changes (Yachi and Loreau, 1999). Our results support prior studies that the rare bacterial taxa have an opportunity to be activated to maintain the stability of the bacterial community under nutrients limitations (Jousset et al., 2017). The snowfall disturbance leads to a decrease in nitrogen content and there is a decrease in temperature at the same time. After the snowfall disturbance, some rare species were adapted to the cold and oligotrophic environment and became abundant species, contributing 22% to the overall community. Four ASVs belonged to *Flavobacterium* (ASV_4, OTU_26, OTU_145, and OTU_146), one ASV belonged to *Psychrobacter* (ASV_9) belong to the rare species in samples before snowfall became abundant after snowfall disturbance (Supplementary Table 3). The genus *Flavobacterium* is known as exopolysaccharide (EPS) producing psychrophiles bacterium. EPS can substantially improve the tolerance of *Flavobacterium* from freeze–thaw cycles to survive in cold regions. When exposed to a cold environment suddenly, a number of physiological changes occur in the cells, and a set of small molecules are synthesized. The genes encoding these cold-shock-inducible proteins, such as cold shock protein (*cspA*), have been identified in *Flavobacterium* and *Psychrobacter* strains (Liu et al., 2019). They have been frequently reported in oligotrophic Antarctic freshwater systems (Michaud et al., 2012), Antarctic cryoconite holes from Canada Glacier (Christner et al., 2003), and Svalbard soils (Hansen et al., 2007), thus suggesting their bloom in oligotrophic and cold conditions after the snowfall.

## Conclusion

This study explored the response of supraglacial cryoconite bacterial communities to a snowfall disturbance at the Laohugou Glacier (Tibetan Plateau). Our results showed that nitrogen concentration plays an important role in shaping the bacterial community diversity and structure in Tibetan glacial cryoconite. After the disturbance of a snowfall, the contribution of deterministic processes significantly increased, and the bacterial community assembly process changes were mainly caused by environmental filtration and bio-filtrating. Our results also revealed that some cold-tolerant rare taxa became abundant in the community after snowfall disturbance. Our results highlight the importance of rare taxa in maintaining glacial biodiversity and ecosystem stability. In addition, these results reveal that, from an ecological perspective, assembly processes play an important role in shaping the cryoconite bacterial community in Tibetan Plateau.

## Data Availability Statement

The datasets presented in this study can be found in online repositories. The names of the repository/repositories and accession number(s) can be found below: https://www.ncbi.nlm.nih.gov/bioproject/PRJNA509012.

## Author Contributions

YQL designed the study and revised the manuscript. YC performed the lab experiments and wrote the draft. YL sampled the cryoconite from the Laohugou glacier. YC, KL, and MJ analyzed the data. All authors approved the final version.

## Conflict of Interest

The authors declare that the research was conducted in the absence of any commercial or financial relationships that could be construed as a potential conflict of interest.

## Publisher’s Note

All claims expressed in this article are solely those of the authors and do not necessarily represent those of their affiliated organizations, or those of the publisher, the editors and the reviewers. Any product that may be evaluated in this article, or claim that may be made by its manufacturer, is not guaranteed or endorsed by the publisher.

## Footnotes

1
http://129.15.40.240/mena/
