# Supplementary material for: Snowstorm Enhanced the Deterministic Processes of the Microbial Community in Cryoconite at Laohugou Glacier, Tibetan Plateau
Source: Front Microbiol. 2022 Jan 27;12:784273. doi: 10.3389/fmicb.2021.784273 (PMC8829297; doi:10.3389/fmicb.2021.784273)
Supplement: Supplementary file 1 [file Table_1.docx]

**Snowstorm enhanced the deterministic processes of the microbial community in cryoconite at Laohugou Glacier, Tibetan Plateau**

**Yuying Chen^1,3^, Yongqin Liu^1,2,3*^, Keshao Liu^1,3^,** **Mukan Ji^2^, Yang Li^4^**

^1^ *State Key Laboratory of Tibetan Plateau Earth System, Resources and Environment (TPESRE), Institute of Tibetan Plateau Research, Chinese Academy of Sciences, Beijing 100101, China*

^2^ *Center for the Pan-third Pole Environment, Lanzhou University, Lanzhou 730000, China*

^3^ *University of Chinese Academy of Sciences, Beijing 100049, China*

^4^ *Institute of International Rivers and Eco-security, Yunnan University, Kunming, Yunnan 650091, China*

* **Correspondence:**

Yongqin Liu

[yqliu@itpcas.ac.cn](mailto:yqliu@itpcas.ac.cn)

Keywords: cryoconite; snowfall; rare bacteria; deterministic processes; stochastic processes

Running title: Microbial community changes in cryoconite

Table S1 General description of the microbial community sequencing.

| Samples | Raw reads | ASVs number | Abundant ASVs number | Rare ASVs number | Oscillating ASVs number |
| --- | --- | --- | --- | --- | --- |
| A-Day1 | 47322 | 627 | 48 | 451 | 128 |
| A-Day4 | 35132 | 590 | 48 | 414 | 128 |
| A-Day8 | 37279 | 576 | 48 | 400 | 128 |
| A-Day26 | 47253 | 566 | 38 | 473 | 55 |
| A-Day31 | 46895 | 307 | 40 | 218 | 49 |
| B-Day1 | 39631 | 624 | 48 | 448 | 128 |
| B-Day4 | 34966 | 559 | 48 | 383 | 128 |
| B-Day8 | 47976 | 595 | 48 | 419 | 128 |
| B-Day26 | 47244 | 586 | 36 | 495 | 55 |
| B-Day31 | 49082 | 256 | 35 | 181 | 40 |
| C-Day1 | 50149 | 588 | 47 | 417 | 124 |
| C-Day4 | 40489 | 530 | 47 | 369 | 114 |
| C-Day8 | 49215 | 609 | 48 | 433 | 128 |
| C-Day26 | 36153 | 542 | 40 | 445 | 57 |
| C-Day31 | 49290 | 315 | 37 | 233 | 45 |
| D-Day1 | 41493 | 587 | 48 | 411 | 128 |
| D-Day4 | 47423 | 506 | 46 | 338 | 122 |
| D-Day8 | 52460 | 505 | 46 | 341 | 118 |
| D-Day26 | 37269 | 570 | 37 | 478 | 55 |
| D-Day31 | 43621 | 241 | 34 | 167 | 40 |

Table S2 The percentage of ASVs changed after snowfall disturbance. None means the rare ASVs not be detected after the snowfall.

| Before | After | Percentage |
| --- | --- | --- |
| Abundant | Abundant | 59% |
| Abundant | Rare | 8% |
| Abundant | Oscillating | 35% |
| Rare | Abundant | 1% |
| Rare | Rare | 93% |
| Rare | Oscillating | 1% |
| Rare | None | 5% |
| Oscillating | Abundant | 2% |
| Oscillating | Rare | 69% |
| Oscillating | Oscillating | 29% |

Table S3 Information of abundant ASVs turns from rare and oscillating bacterial taxa.

| ASV name | | Before snowfall | | Relative abundance (%) | After snowfall | Relative abundance (%) | | Phylum | | Class | | Order | Family | Genus |
| --- | --- | --- | --- | --- | --- | --- | --- | --- | --- | --- | --- | --- | --- | --- |
| ASV_124 | | Rare | | 0.02 | Abundant | 0.35 | | Proteobacteria | | *Betaproteobacteria* | | *Burkholderiales* | *Oxalobacteraceae* | *Massilia* |
| ASV_145 | | Rare | | 0.03 | Abundant | 0.23 | | Bacteroidetes | | *Flavobacteriia* | | *Flavobacteriales* | *Flavobacteriaceae* | *Flavobacterium* |
| ASV_146 | | Rare | | 0.04 | Abundant | 0.20 | | Bacteroidetes | | *Flavobacteriia* | | *Flavobacteriales* | *Flavobacteriaceae* | *Flavobacterium* |
| ASV_26 | | Rare | | 0.01 | Abundant | 2.22 | | Bacteroidetes | | *Flavobacteriia* | | *Flavobacteriales* | *Flavobacteriaceae* | *Flavobacterium* |
| ASV_4 | | Rare | | 0.02 | Abundant | 12.52 | | Bacteroidetes | | *Flavobacteriia* | | *Flavobacteriales* | *Flavobacteriaceae* | *Flavobacterium* |
| ASV_75 | | Rare | | 0.00 | Abundant | 0.61 | | Proteobacteria | | *Gammaproteobacteria* | | *Pseudomonadales* | *Moraxellaceae* | *Psychrobacter* |
| ASV_88 | | Rare | | 0.03 | Abundant | 0.54 | | Actinobacteria | | *Actinobacteria* | | *Actinomycetales* | *Cellulomonadaceae* | *Cellulomonas* |
| ASV_9 | | Rare | | 0.00 | Abundant | 5.57 | | Proteobacteria | | *Gammaproteobacteria* | | *Pseudomonadales* | *Moraxellaceae* | *Psychrobacter* |
| ASV_98 | | Rare | | 0.00 | Abundant | 0.48 | | Firmicutes | | *Bacilli* | | *Lactobacillales* | *Carnobacteriaceae* | *Carnobacterium* |
| ASV_2 | | Oscillating | | 0.08 | Abundant | 17.91 | | Bacteroidetes | | *Flavobacteriia* | | *Flavobacteriales* | *Flavobacteriaceae* | *Flavobacterium* |
| ASV_57 | Oscillating | | 0.15 | | Abundant | | 0.89 | | Proteobacteria | | *Betaproteobacteria* | *Burkholderiales* | *Comamonadaceae* | *Polaromonas* |
| ASV_90 | Oscillating | | 0.09 | | Abundant | | 0.51 | | Proteobacteria | | *Betaproteobacteria* | *Burkholderiales* | *Comamonadaceae* | *Polaromonas* |


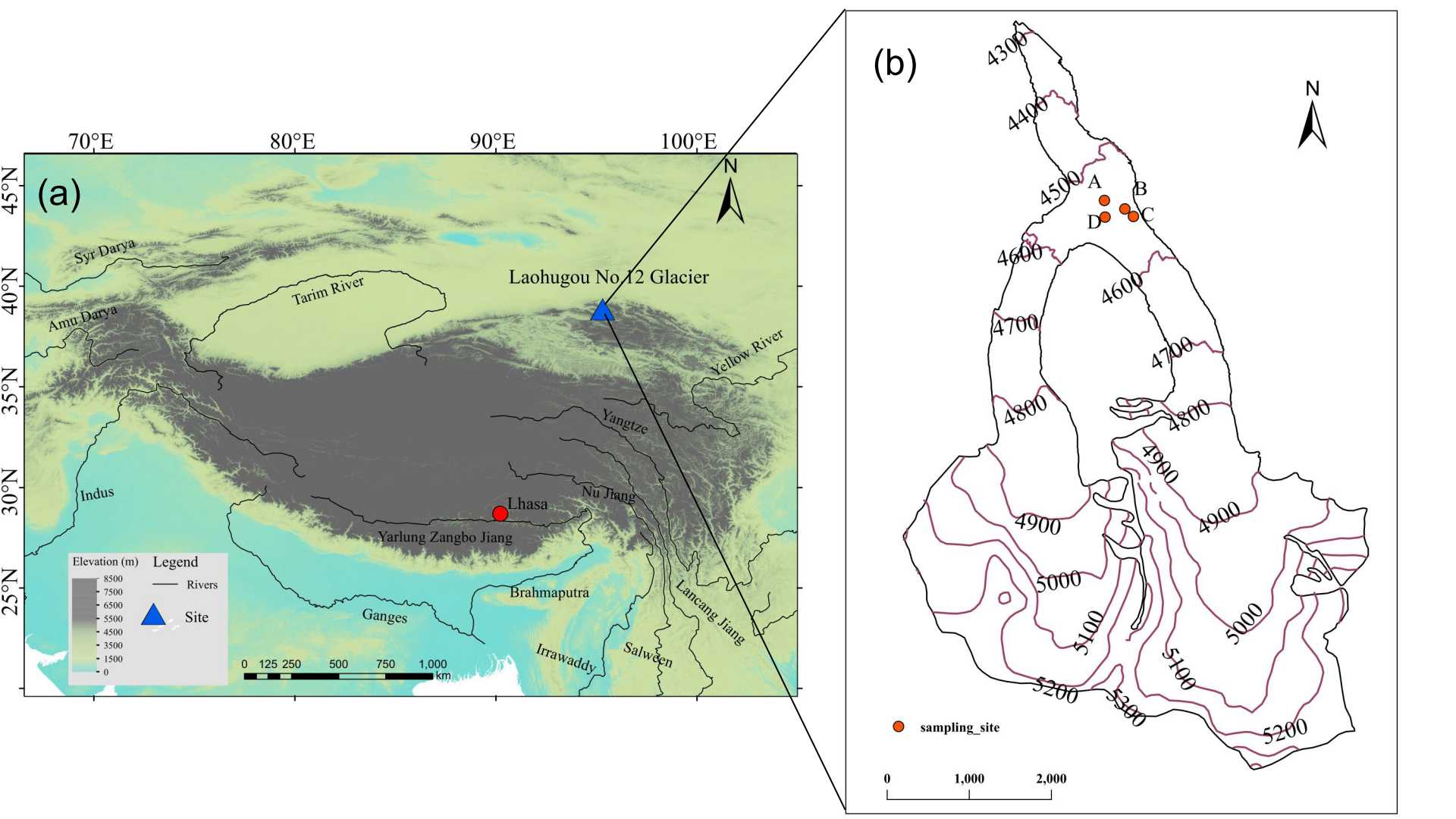


Figure S1 (a) Topographic map of Laohugou No.12 glacier in the northeast of Tibetan Plateau, with (b) the sampling sites marked from A-D.


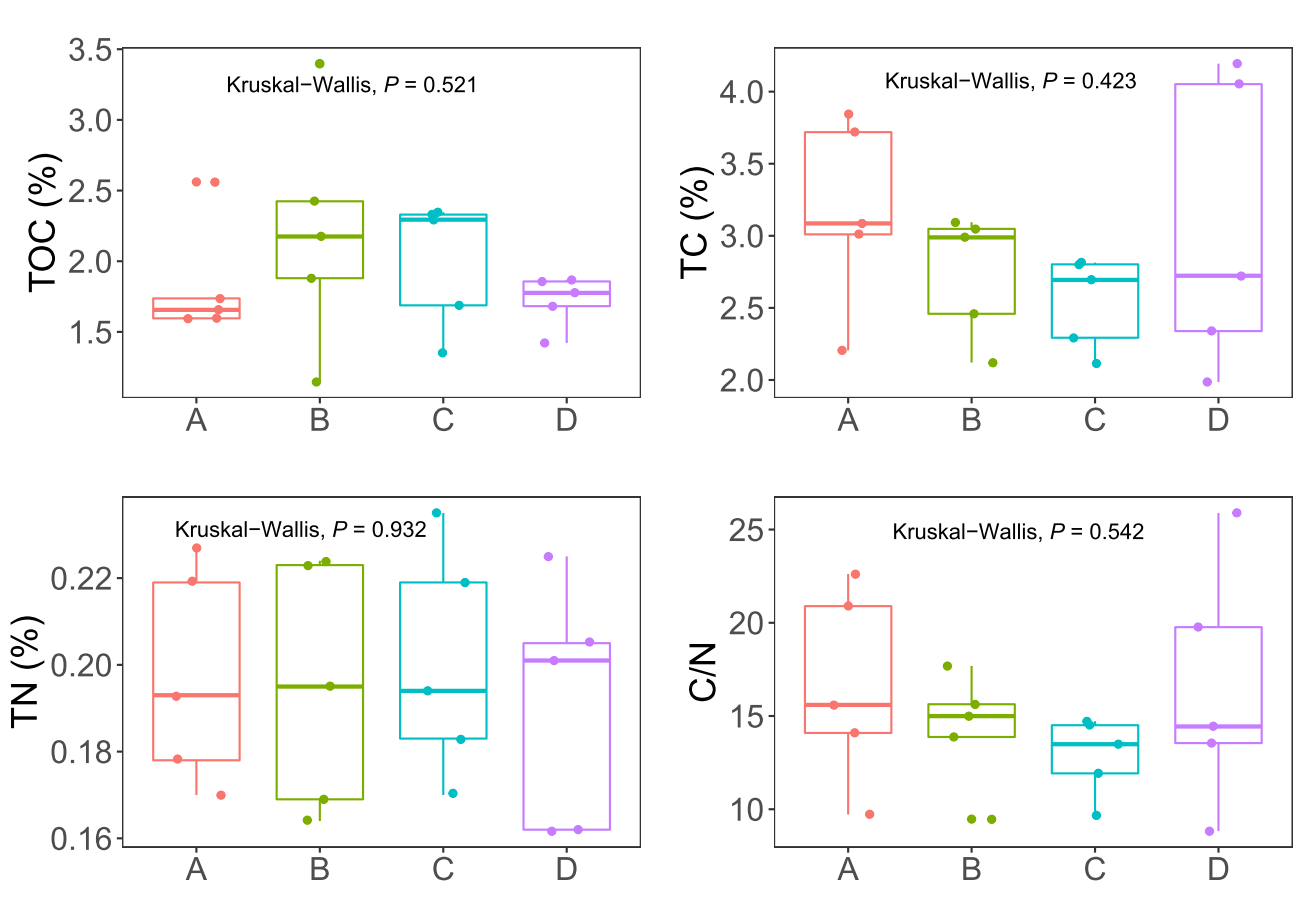


Figure S2 Variations in environmental characteristics between different sampling sites. All environmental characteristics showed no significant difference between sampling sites. Comparison is based on Kruskal-Wallis test.


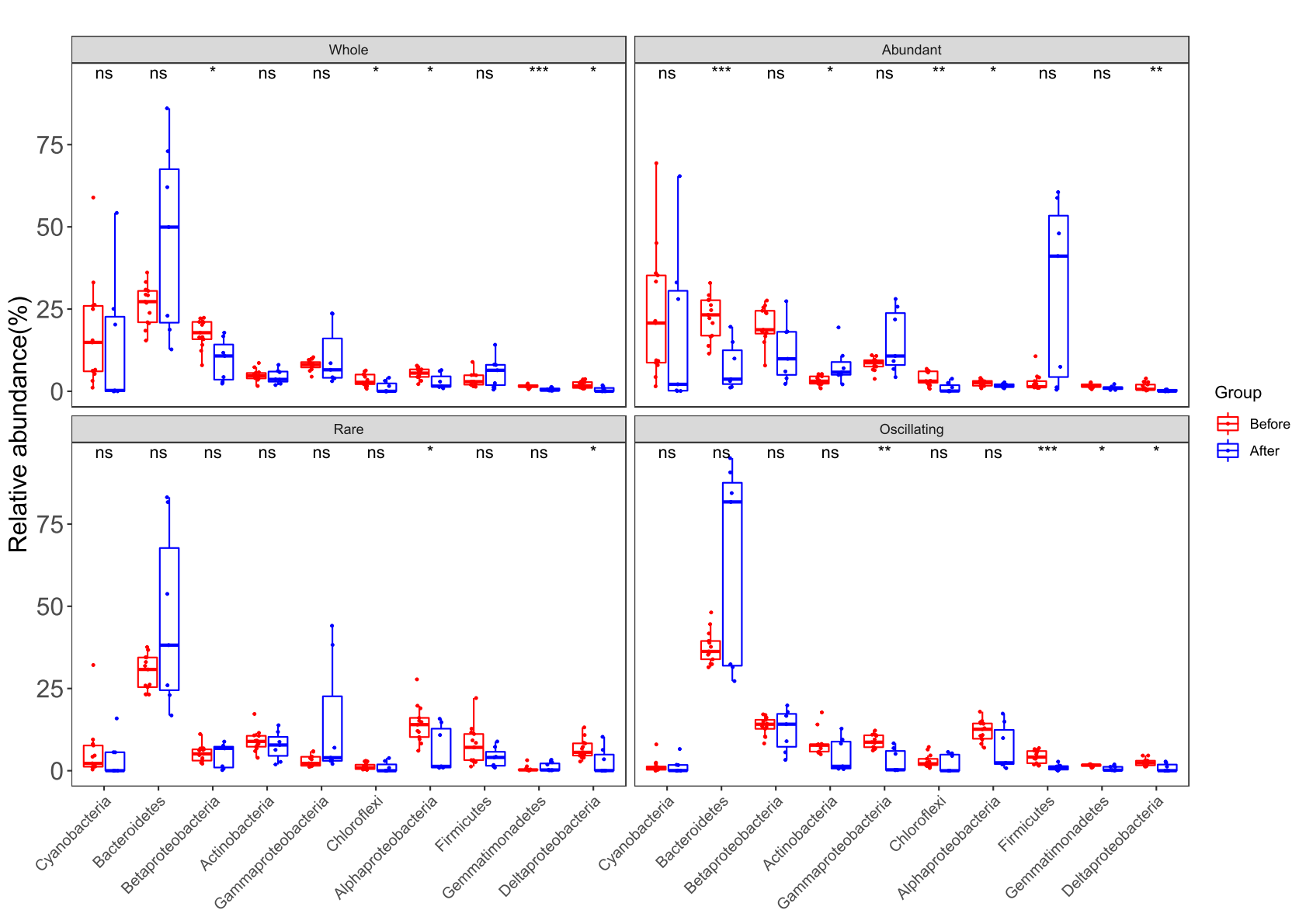


Figure S3. Relative abundances of the dominant bacterial phyla/classes in before and after snowfalls in whole, abundant, and rare communities. Comparison is based on Wilcoxon rank-sum test. ∗ *P* < 0.05, ∗∗ *P* < 0.01, ∗∗∗ *P* < 0.001, ns *P* > 0.05.


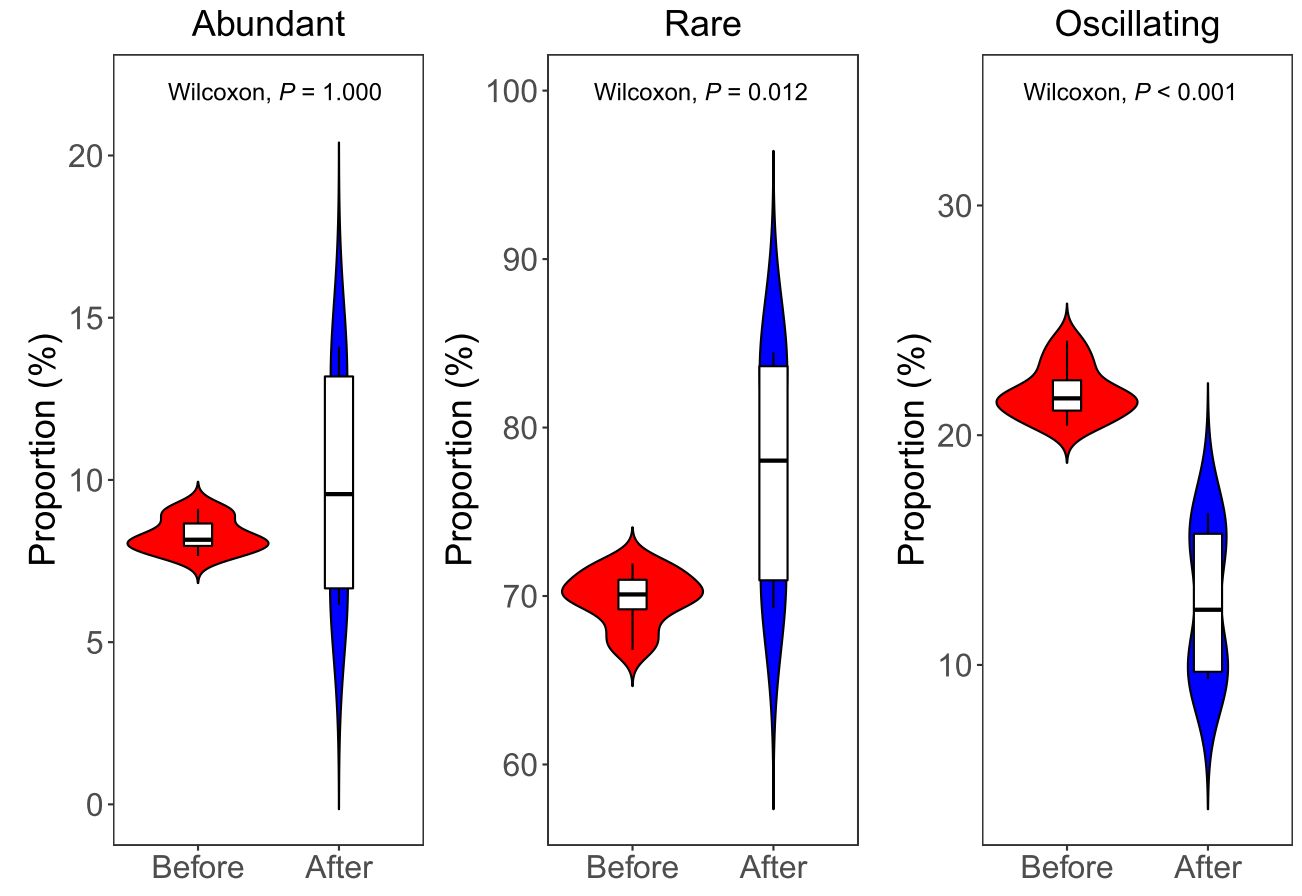


Figure S4 The proportion of abundant, rare, and oscillating bacterial taxa. A significantly higher proportion of rare taxa was observed in the after snowfall samples based on Wilcoxon rank-sum test, while a significantly higher proportion of oscillating taxa was observed in the before snowfall samples based on Wilcoxon rank-sum test.

Taxonomic compositions

and abundance-occupancy rela-

tionships. The taxonomic compo-

sitions of rare, abundant and

entire bacterial communities are

shown in (A), and the relative

abundance–occupancy relation-

ships of rare and abundant OTUs

are shown in (B).

Taxonomic compositions

and abundance-occupancy rela-

tionships. The taxonomic compo-

sitions of rare, abundant and

entire bacterial communities are

shown in (A), and the relative

abundance–occupancy relation-

ships of rare and abundant OTUs

are shown in (B).


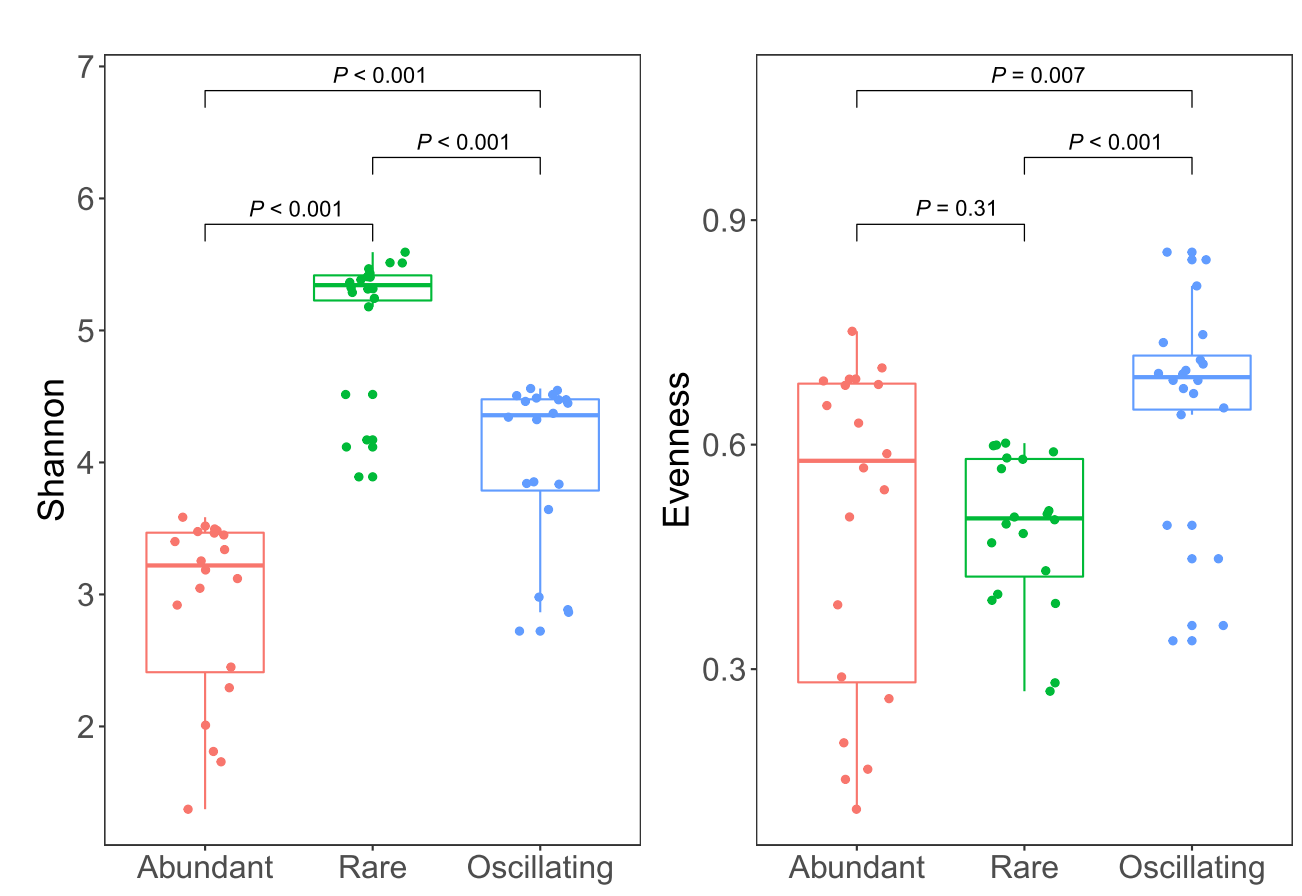


Figure S5 Bacterial alpha diversity comparison between the abundant, rare and oscillating subcommunities. Each dot represents an individual sample. For Shannon indices, the rare bacterial taxa are significantly higher than the rare and oscillating bacterial taxa. Comparison is based on Wilcoxon rank-sum test.
